# Anxiety symptoms interact with approach motivations in adolescent risk-taking

**DOI:** 10.1017/S0954579426101266

**Published:** 2026-02-24

**Authors:** Amanda E. Baker, Namita Tanya Padgaonkar, Isabel Enriquez, Tara S. Peris, Adriana Galván

**Affiliations:** 1 Department of Psychology, University of California Los Angeles, Los Angeles, CA, USA; 2 Department of Psychiatry and Biobehavioral Sciences, University of California Los Angeles, Los Angeles, CA, USA; 3 Division of Child and Adolescent Psychiatry, UCLA Semel Institute for Neuroscience and Human Behavior, Los Angeles, CA, USA

**Keywords:** Anxiety, Approach, Adolescence, Risk-taking, fMRI

## Abstract

Adolescence represents a pivotal neurodevelopmental period marked by escalating anxiety symptoms and heightened approach motivations. Although anxiety is typically linked to avoidance, concurrent shifts in motivational systems and neurocircuitry may alter its behavioral and neural expression, shaping developmental trajectories and treatment response. This study investigated how approach motivations (Behavioral Activation System; BAS) interact with anxiety to influence behavior and brain function in *N* = 121 adolescents (ages 9–13; 44% girls; 33.1% White, 22.3% Latino, 19.8% Asian, 14.9% Black, 9.9% Mixed Race). Participants completed a decision-making task and resting-state fMRI. Dimensional analyses examined joint effects of anxiety and BAS on risk-taking behaviors, task-evoked neural activity and connectivity, and intrinsic connectivity at rest. Higher anxiety was associated with risk aversion and inhibition when BAS was low, but with risk-taking and impulsivity when BAS was high (risk-taking: *β* = 0.25, *p* = .012; inhibitory control: *β* = 0.13, *p* < .001). During risk-taking, anxiety and BAS showed interactive effects on striatal (*β* = −0.10, *p* = .006) and amygdala (*β* = 0.10, *p* = .005) activity alongside distinct effects on prefrontal–subcortical connectivity (*β* = −0.30, *p* = .014; *β* = 0.17, *p* = .01). Higher BAS was associated with stronger intrinsic prefrontal–striatal connectivity (*β* = 0.23, *p* = .012), while anxiety showed no significant resting-state effects. Findings underscore the role of reward-related systems in adolescent anxiety and support developmentally informed, personalized intervention strategies.

## Introduction

Adolescence is a pivotal developmental period marked by rapid neurobiological and socioemotional changes that reshape how youth respond to reward, threat, and uncertainty (Casey et al., 2008; Ernst et al., 2009). During this window, frontolimbic circuits – including the amygdala, ventral striatum (VS), and dorsal anterior cingulate cortex (dACC) – undergo extensive reorganization, with age-related changes in activation and connectivity patterns supporting increasingly flexible emotion regulation and goal-directed behavior (Ahmed et al., 2015; Insel et al., 2017; Poon et al., 2022; Stephanou et al., 2016; Wilbrecht & Davidow, 2024). These shifts contribute to adaptive increases in approach behavior and risk-taking that support exploration and learning (Casey et al., 2008), while also rendering adolescence a period of heightened vulnerability for the onset and intensification of clinical anxiety (Gee et al., 2016). This creates a developmental context in which avoidance tendencies must be understood alongside rising approach-driven motivation. Critically, heightened neural plasticity in early adolescence creates a window of opportunity in which regulatory circuits are still forming and behavioral patterns are not yet entrenched (Baker et al., 2025). Understanding how threat and reward systems interact during this period is essential for identifying mechanisms that may shape long-term anxiety trajectories before maladaptive patterns consolidate.

Contemporary models of adolescent anxiety emphasize that anxiety rarely reflects pure avoidance; instead, it often involves simultaneous activation of approach and threat-avoidance systems, resulting in motivational conflict (Aupperle & Paulus, 2010; Baker & Galván, 2020; Barker et al., 2019; Letkiewicz et al., 2023). From this perspective, anxious youth may be pulled toward rewarding or goal-oriented behaviors while also experiencing heightened sensitivity to potential threat. This conflict may be especially relevant during adolescence, a period characterized by greater reward seeking, risk tolerance, and exploration alongside still-maturing prefrontal regulatory systems (Casey et al., 2008; Tymula et al., 2012). During this time, individual differences in approach motivations may fundamentally shape the behavioral expression of anxiety, amplifying or buffering risk-related behavior depending on the adolescent’s motivational profile.

Consistent with this view, research investigating the frequent co-occurrence of anxiety and substance abuse in adults suggests that there may be two motivationally distinct subtypes: an “avoidant subtype” characterized by behavioral inhibition and risk aversion, and an “approach-motivated subtype” exhibiting impulsivity, reward sensitivity, and risk-taking (Nicholls et al., 2014). Adolescence may amplify these distinctions, making motivational heterogeneity particularly important to understanding symptom presentation and functional outcomes.

Motivational heterogeneity also carries clinical significance. Youth with high approach tendencies may be more prone to impulsive risk-taking or substance use despite elevated anxiety, whereas low-approach anxious youth may display more withdrawal-based profiles. Differentiating these phenotypes in early adolescence may help refine prevention and personalize treatment strategies, particularly as sensitivity of the behavioral activation system (BAS) – reflecting approach motivations and reward-related traits – predicts cognitive behavioral therapy (CBT) engagement and outcomes in anxious youth (Norris et al., 2021).

These ideas also align with the Triadic Model of adolescent motivated behavior, which proposes that heightened subcortical reactivity (VS and amygdala) combined with still-developing prefrontal control biases adolescents toward approach behavior (Ernst, 2014; Ernst et al., 2006). Within this framework, approach and avoidance processes are not independent but dynamically co-activated, and motivational conflict arises when strong reward-driven tendencies coincide with increased threat sensitivity. Because subcortical systems are highly responsive and regulatory prefrontal circuitry is still maturing during early adolescence (Casey et al., 2008), this developmental stage may be especially susceptible to the kinds of motivational competition that characterize many presentations of adolescent anxiety. Integrating the Triadic Model with approach–avoidance conflict accounts suggests that anxious adolescents with higher approach motivations may experience amplified competition between reward-driven and avoidance-driven signals – yielding heterogeneous patterns of risk-taking and neural recruitment which could impact symptom presentation and progression. From a clinical perspective, this competition between reward-driven and threat-avoidant processes may create distinct pathways to impairment – one marked by excessive avoidance, another by dysregulated approach despite anxiety. Identifying which adolescents fall into which trajectory during this formative period could substantially improve early identification and intervention.

These motivational dynamics align closely with ongoing changes in neural circuits involved in evaluating and regulating reward and threat. Early adolescence – the window targeted in this study – is marked by substantial development connectivity between the amygdala and the regulatory prefrontal cortex (PFC), with typical trajectories involving a shift from positive to inverse (regulatory) coupling (Gee et al., 2013). Striatal responsivity to reward and prefrontal conflict-monitoring processes also undergo refinement during this period (Galvan et al., 2006; Galván, 2013; Insel et al., 2017). Deviations from these normative trajectories, such as persistent positive amygdala–PFC coupling, are linked to heightened anxiety and difficulty disengaging from threat (Gee et al., 2013).

The dACC is particularly relevant to these interactions, given its role in conflict monitoring and in integrating information about reward and threat during value-based decision-making (Christopoulos et al., 2009; Kolling et al., 2014; B. W. Smith et al., 2009). Reduced dACC engagement is observed in anxious adults during emotion regulation (Blair et al., 2012), and anxious adolescents who show blunted dACC responses to social exclusion are more likely to develop substance use problems (Beard et al., 2022). As dACC-subcortical circuits continue to mature through adolescence, age-related variability in their development may help explain individual differences in how approach motivations and anxiety jointly shape risk-taking.

Despite strong theoretical grounding, no study has systematically examined how anxiety and approach motivations jointly influence adolescent risk-taking or how these interactions are instantiated across functioning of amygdala, VS, and dACC circuits during risk-taking and at rest. Clarifying these mechanisms is essential for identifying motivationally distinct anxiety profiles and for developing biologically informed intervention strategies. Because early adolescence is a period when anxiety symptoms often first emerge and neural circuits remain malleable, clarifying these mechanisms now is critical for informing developmental models of risk and improving early, targeted intervention efforts.

The present study addresses these gaps using behavioral and fMRI data from a large community sample of early adolescents (ages 9–13). This age range captures a window of rapid subcortical–prefrontal development, heightened approach behavior, and emerging anxiety symptoms, when interactions between motivational drives and threat sensitivity may have especially strong developmental consequences.

Aim 1 tests whether sensitivity of the BAS moderates the association between anxiety and behavior on a risky decision-making task. Pre-registered hypotheses for Aim 1: As the approach-motivated anxiety subtype has been characterized by impulsivity and risk-taking, we predicted increasing anxiety would be associated with greater risk-taking and slower inhibitory control in the context of high BAS, while increasing anxiety would be associated with risk aversion and faster inhibition in the context of low BAS.

Aim 2 examines how BAS and anxiety relate to functioning of the amygdala, VS, and dACC during risk-taking. Pre-registered hypotheses for Aim 2: As anxious youth show heightened amygdala activation during risk-taking (Galván & Peris, 2014) and amygdala-PFC connectivity to emotional images (Poon et al., 2022), we predicted anxiety would correlate positively with amygdala response and amygdala-dACC connectivity during risk-taking. As BAS has been linked to heightened VS response during reward receipt (Mohammadzadeh Ebrahimi et al., 2015), we predicted that adolescents with high BAS would show heightened VS response paralleled by reduced VS-dACC functional connectivity during risk-taking. We also conducted exploratory interaction analyses to examine whether behavioral patterns involving anxiety and approach motivations extend to brain function during risk-taking.

Aim 3 examines how BAS and anxiety relate to intrinsic connectivity of the amygdala, VS, and dACC at rest. Examining resting-state in conjunction with task fMRI allows us to measure neural function across domains and help clarify the scope of influence of individual difference measures on adolescent brain function (i.e., which neural markers are specific reactions to decision-making vs. which are stable markers that persist when the brain is at rest). Pre-registered hypotheses for Aim 3: As anxious adults demonstrate reduced intrinsic amygdala-PFC connectivity (Kim et al., 2011), we predicted reduced intrinsic amygdala-dACC connectivity in youth with higher anxiety. As BAS has been linked to heightened intrinsic connectivity between the striatum and the orbitofrontal cortex in adults (Angelides et al., 2017), we predicted that adolescents with high BAS would show heightened fronto-striatal connectivity at rest. Additionally, we conducted exploratory interaction analyses to examine whether behavioral patterns involving anxiety and approach motivations extend to intrinsic brain function during resting-state. Given known age-related shifts in intrinsic amygdala–PFC connectivity (Gee et al., 2013), we also conducted exploratory age-by-anxiety interaction analyses to probe whether developmental stage moderated these associations.

Exploratory Aim: Individual differences can bias behavior towards excessive avoidance, forgoing potential rewarding experiences, or excessive approach, ignoring potential harm. Both positive and aversive biases are linked to dopaminergic receptors in the VS (Nguyen et al., 2018), which helps explain why adolescent VS sensitivity has been implicated in both anxiety (Lago et al., 2017) and risk-taking (Chein et al., 2011). We conducted an exploratory analysis to probe how the adolescent striatum biases behavior towards or away from risk using representational similarity analysis (RSA) (Kriegeskorte et al., 2008) that involves leveraging information contained in the *patterns* of activity across multiple voxels of the brain to characterize how the brain distinguishes between or generalizes across stimuli. This approach has helped delineate how anxious youth differentiate between threat and safety (Glenn et al., 2020) and therefore seems a promising approach for parsing how striatal connectivity patterns encode risky and cautious decisions in adolescence. Although this analysis was not preregistered, we predicted that adolescents with higher anxiety would show more striatal differentiation between risky and cautious decisions, suggesting that the striatum may be uniquely tuned to assessing safety versus risk in higher anxiety, while adolescents with high BAS would show more striatal generalization across risky and cautious decisions, indicating an approach-related bias in contexts of potential risk and reward.

## Method

### Participants

We recruited 171 youth (ages 9–13 years) from a large metropolitan area to complete a clinical interview and fMRI scan. Recruitment was designed to capture a broad range of anxiety symptom severity, with targeted outreach strategies (e.g., community flyers, school partnerships, clinic-based referrals) intended to oversample youth with elevated anxiety symptoms just below clinical thresholds. The full sample completed the Screen for Child Anxiety Related Emotional Disorders (SCARED; Birmaher et al., 1997) as a dimensional measure of anxiety severity. SCARED scores were not used for selection but provided a continuous index of symptom variability. Although SCARED scores were not used as a formal eligibility screen, they were used to quantify individual differences in anxiety severity across the full spectrum – from low to high symptoms.

Inclusion criteria were: right-handed, fluent in English, no MRI contraindications (e.g., metal implants, claustrophobia), and no current use of psychotropic medications. Youth were excluded for neurological (e.g., seizures, stroke) or developmental (e.g., autism) conditions, current suicidality, major depressive disorder, or severe psychiatric conditions (e.g., schizophrenia, substance use disorder) requiring pharmacologic treatment or likely to obscure anxiety-specific effects. Diagnostic status was determined via the Anxiety Disorders Interview Schedule for DSM-IV (ADIS-IV; Silverman & Albano, 1996), administered by a trained clinician.

Of the 171 participants, 121 had usable task fMRI data (*M*_Age_ = 11.24, *SD*_Age_ = 1.37; Table 1a) and 124 had usable resting-state data (*M*_Age_ = 11.30, *SD*_Age_ = 1.43; Table 1b). Twenty-five youth did not complete the scan (13 due to COVID-related cancellations; 12 due to discomfort in the MRI environment). Six participants were excluded from task analyses and 5 from resting-state analyses due to missing BAS data. Task data from 5 additional youth were excluded due to technical issues, and 14 were excluded for insufficient trials. Resting-state data were excluded for 5 participants based on motion thresholds (≥1 mm mean relative or ≥5 mm absolute displacement). Mean relative motion was low across both sequences (task: *M* = 0.24 mm, *SD* = 0.15; rest: *M* = 0.23 mm, *SD* = 0.15) and was not significantly correlated with anxiety or BAS in either modality. Similarly, logistic regression indicated that neither SCARED scores nor BAS scores predicted missingness in neuroimaging data in either modality (driving game: *β*_SCARED_ = 0.005, *p* = .84, *β*_BAS_ = 0.044, *p* = .42; resting-state: *β*_SCARED_ = –0.023, *p* = .44, *β*_BAS_ = –0.011, *p* = .84).

**Table 1.** Participant descriptive statistics for (a) task fMRI and (b) resting state. SD = standard deviation; BAS = behavioral activation system; RT = response time

Task fMRI	Mean (SD) or % (*N* = 121)
Age (years)	11.19 (1.36), range = 9–13
Sex	68M (56.2%), 53F (44%)
Race/ethnicity	33.1% white, 22.3% Latino, 19.8% Asian, 14.9% Black, 9.9% Mixed Race
IQ (WASI-II Vocab and Matrix Reasoning)	109.81 (17.8), range = 61–155, skewness = −0.06
Anxiety (SCARED total score)	19.0 (11.4), range = 0–52, skewness = 0.64
BAS total score	38.73 (5.2), range = 26–52, skewness = −0.29
Risk-taking frequency	14.41 (7.7), range = 3–32, skewness = .41
Inhibition RT (seconds)	0.59 (0.01), range = 0.34–0.81, skewness = 0.07
Go RT (seconds)	0.46 (0.01), range = 0.30–0.69, skewness = 0.35
Inhibition–Go RT	0.12 (0.07), range = −0.18–0.29, skewness = −0.55
Mean relative motion (mm)	0.23 (0.13)
**Resting state**	**Mean (SD) or % (*N* = 124)**
Age (years)	11.39 (1.43)
Sex	64M (51.6%), 60F (48.4%)
Race/ethnicity	33.1% white, 24.2% Latino, 20.2% Asian, 13.7% Black, 8.9% Mixed Race
IQ (WASI-II Vocab and Matrix Reasoning)	110.72 (17.83), range = 61–155, skewness = −0.09
Anxiety (SCARED total score)	19.3 (10.98), range = 0–52, skewness = 0.51
BAS total score	39.89 (5.12), range = 26–52, skewness = −0.18
Mean relative motion (mm)	0.24 (0.15)

Seven of the 121 participants included in the task sample met diagnostic criteria for an anxiety disorder, 5 met criteria for comorbid anxiety and ADHD, 12 were diagnosed with a non-anxiety disorder (e.g., ADHD, OCD, tic disorder), and 97 did not meet criteria for any disorder. Diagnoses were similar in the resting-state sample (8 anxiety, 6 comorbid, 10 other, 100 none). Participants on ADHD medications completed a 48-hour washout before scanning.

### Procedures

Youth and caregivers completed two in-person study visits. At Visit 1, they completed questionnaires assessing anxiety symptoms and approach–avoidance motivations. Clinicians administered the ADIS-IV to assess diagnostic status. At Visit 2, participants underwent an MRI scan including both a task-based fMRI paradigm (Driving Game; see below) and an 8-minute resting-state scan. A mock scanner was used for acclimation. Youth received $100 for participation and could earn an additional $10 based on task performance.

### Ethical considerations

Informed consent and assent were obtained from all legal guardians and study participants in accordance with the Institutional Review Board.

### Measures

#### Dimensional anxiety severity

Participants completed the 41-item self-report SCARED as a dimensional measure of anxiety severity. They rated statements describing anxiety symptoms (e.g., “I feel nervous around people I don’t know very well”) on a 3-point Likert scale from 0–2 (*Not True/Hardly Ever True*-*Very True/Often True*).

#### Approach/avoidance motivations

Participants rated approach-related (e.g., “I go out of my way to get things I want”) and avoidance-related (e.g., “I worry about making mistakes”) statements on the Behavioral Inhibition System/Behavioral Activation System (BIS/BAS) scales (Carver & White, 1994) on a 4-point Likert scale from 1–4 (*very true for me*-*very false for me*). Ratings were summed across the 3 approach-related subscales (Drive, Fun Seeking, Reward Responsiveness) into a BAS total score. We tested the bivariate correlation between anxiety (SCARED total score) and approach motivations (total BAS score), finding no significant association (*r* = 0.08, *p* = 0.41), suggesting that these constructs are not strongly correlated in this sample and can be examined as independent predictors in moderation analyses.

### Task fMRI paradigm

**Figure 1.**
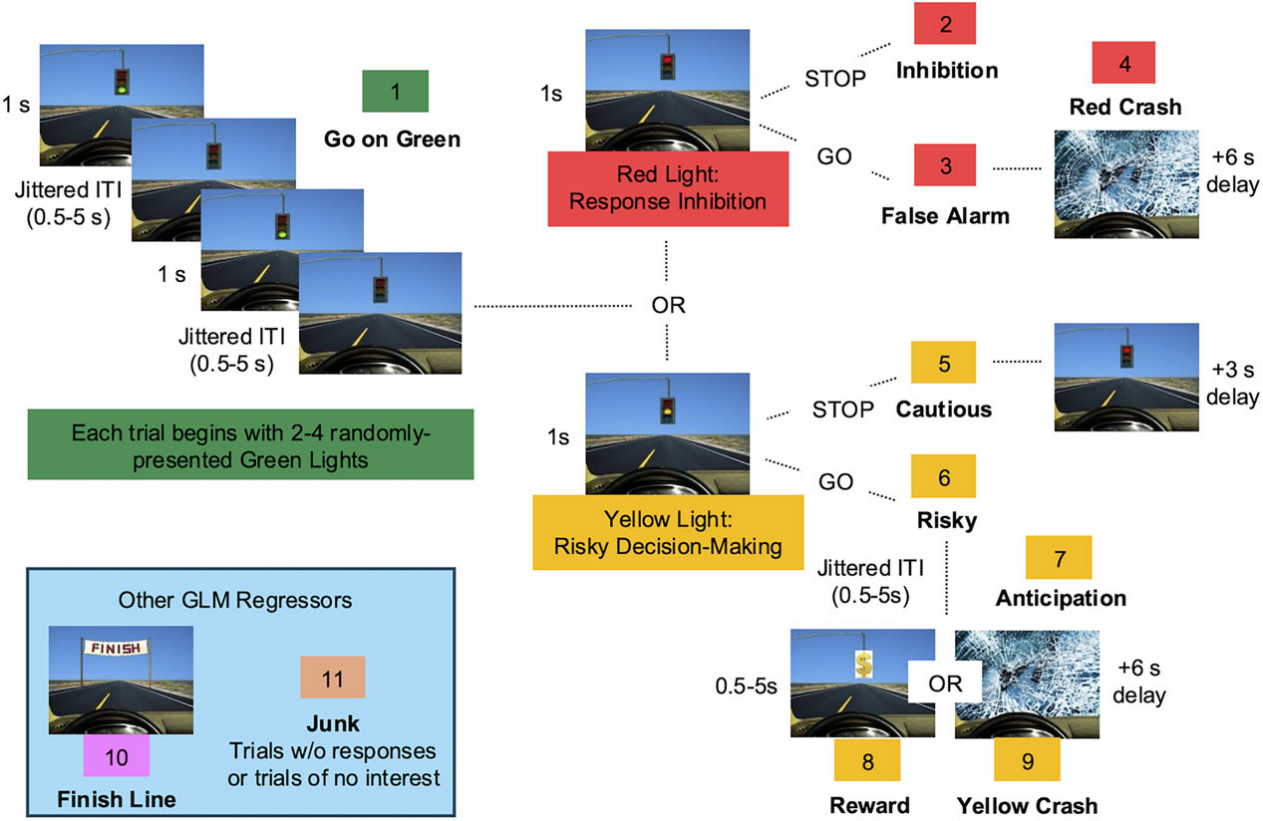
The Driving Game. Participants encountered green, red, and yellow stoplights in the laboratory task and were instructed to press “1” to go at green, “2” to stop at red, and either “1” to go (Risky) or “2” to stop (Cautious) at yellow lights. A jittered inter-trial (ITI) stimulus followed each event. Trials began with 2–4 green lights and ended with either a red or yellow light (50/50 chance). A risky choice was followed by a 50/50 chance of a reward, reaching the finish line faster and earning more money, or a crash (+6 s delay). Each regressor included in the general linear model (GLM) is indicated by name and number.

Participants played two 8-minute runs of the Driving Game, an adapted version of the Stoplight Task (Chein et al., 2011) involving decision-making at randomly-presented traffic lights and trying to finish quickly to maximize monetary reward (Figure 1). Lights are presented for 1s or until the participant responds and followed by a jittered inter-trial interval (ITI; .5-5s). On each trial, participants were instructed to press “1” to go at a series of green lights, thereby building a prepotent response. Following the green lights, participants encountered a red light or a yellow light (50/50 chance). At a red light, participants were instructed to press “2” to stop the car; failure to stop resulted in a crash (+6s). At a yellow light, participants could choose to press “1” to go (risky choice) or press “2” to stop (cautious choice). A cautious choice led to the light turning red (+3s). A risky choice led to a 50/50 chance of a safe crossing, resulting in a reward and reaching the finish line faster, or a crash, adding 6 s to their route. RT was measured as the duration in milliseconds from stimulus onset to participant response.

The total number of risky choices at yellow lights was used to index risk-taking frequency for each participant. To index inhibitory control for each participant, we used the stop-signal reaction time (SSRT), or the time it takes to inhibit a prepotent response compared to each participant’s Go RT, a commonly used metric of response inhibition amongst developing youth (Cohen et al., 2010). Risk-taking frequency and inhibitory control were correlated (*r*(121) = .35, *p* < .001), suggesting that these two facets of adolescent behavior are intertwined and most likely involve similar processes. As they were measured using the same task, these constructs may show stronger associations here than they would if behavior was compared across different tasks.

Although the Driving Game includes rewards (e.g., faster progress) and penalties (e.g., time delays), it does not include actual monetary contingencies. However, participants were not informed of this. Instead, they were told they could earn a bonus of up to $10 during the MRI visit. Half of this bonus was determined based on performance on a separate probabilistic reward-learning task, and the other half was assigned randomly after completing the Driving Game. This approach ensured that participants engaged with the Driving Game as if reward outcomes were monetarily consequential, maintaining ecological validity without introducing confounds related to actual performance-based payment.

### Resting-state paradigm

Following the task runs, participants completed an 8-minute resting-state fMRI scan. During this scan, they were instructed to lie still with their eyes open and focus on a white fixation cross presented at the center of a black screen. No stimuli were presented, and participants were told to let their minds rest without falling asleep.

### fMRI acquisition

A 20-channel head coil was used on a 3-Tesla Siemens Trio MRI machine. Participants completed a mock scan and were screened with a metal detector before scanning. The task was presented on E-Prime. A Magnetization-Prepared Rapid Gradient-Echo (MPRAGE) scan (TR = 1900 ms, TE = 2.26 ms, FOV = 250 mm, 176 slices, slice thickness = 1 mm, voxel size = 1.0×1.0 mm, interleaved) was used for registration. For B0 distortion correction, participants received 2 T2*-weighted gradient-echo field map scans with opposite phase encoding directions (AP, PA; TR = 8,000 ms, TE = 66 ms, FOV = 208 mm, 72 slices, slice thickness = 2 mm, voxel size = 2×2 mm, interleaved). Two runs of the T2*-weighted task fMRI sequence (TR = 800 ms, TE = 37 ms, FOV = 208 mm, 72 slices, slice thickness = 2 mm, voxel size = 2×2 mm, interleaved) were acquired during the task. After, participants underwent an 8-minute resting-state fMRI sequence (TR = 800 ms, TE = 37 ms, FOV = 208 mm, 72 slices, slice thickness = 2 mm, voxel size = 2×2 mm, interleaved) during which they were instructed to lie still and relax with their eyes focused on a white fixation cross on a black screen. A single-band reference (SBRef) image was acquired before each functional sequence.

### fMRI preprocessing

Field-map images were combined using FSL’s topup (Andersson et al., 2003) and used for B0 unwarping in FEAT V6 (FMRIB Software Library; https://fsl.fmrib.ox.ac.uk/fsl/) (S. M. Smith et al., 2004). Preprocessing steps for task fMRI included non-brain removal using BET, high-pass filtering (100-s), and spatial smoothing using a Gaussian kernel of FWHM 5 mm. Rigid body motion correction with 6° of freedom was performed using MCFLIRT. Functional data were registered to the SBRef, to the MPRAGE, and to Montreal Neurological Institute (MNI) stereotaxic space with 12° of freedom using FSL’s nonlinear registration method FNIRT. FSLMotionOutliers (https://fsl.fmrib.ox.ac.uk/fsl/fslwiki/FSLMotionOutliers) detected timepoints corrupted by a high degree of motion (box-plot cutoff = P75 + 1.5*IQR). The resulting confound matrices were entered as regressors of no interest in the general

linear model (GLM), removing the effects of these timepoints.

Resting-state preprocessing included BET, spatial smoothing (5 mm), and motion correction with 6° of freedom using MCFLIRT. Functional data were registered to the SBRef, to the MPRAGE, and to MNI space with 12° of freedom using FNIRT. Data were denoized using Independent Component Analysis (ICA)-based Automatic Removal of Motion Artifacts (Pruim et al., 2015), a highly effective method for addressing head motion (Parkes et al., 2018). Data were next high-pass filtered (100-s), and white matter and cerebrospinal fluid (CSF) masks were created using FSL FAST. Time series were extracted from the subject-specific white matter and CSF masks and regressed out of the brain data using FEAT. The residuals were normalized and registered to standard space for use in further analysis.

### Behavioral analysis

#### Moderation testing

To test whether BAS moderated the association between anxiety and task risk-taking frequency, we fit a multiple linear regression model using the “lm” function in the “stats” package in R (R Core Team, 2023) with task risk-taking frequency as the dependent variable, anxiety, BAS, and the anxiety*BAS interaction as variables of interest, and age and sex as covariates of no interest. To test whether BAS moderated the association between anxiety and inhibitory control, we fit a mixed-effects multilevel model using the ‘lmer’ function in the “lme4” package in R (Bates et al., 2015) with a fixed effect of Condition (1 = Stop, 2 = Go) and random intercepts to account for repeated measures within subjects. Upon adding a random effect for the slope of Condition, the model with both random slopes and random intercepts demonstrated better model fit (χ^2^ = 284.45, *p* < .001), so both random effects were included. We next added fixed-effects terms for anxiety, BAS, age, and gender to the model, as well as the anxiety *BAS*Condition interaction. Interactions are depicted using -1 SD, mean, and + 1 SD as moderator values (Aiken & West, 1991). Continuous predictors (e.g., anxiety, BAS, age) were standardized (*z*-scored) using the base R scale() function prior to inclusion in the models.

### fMRI analysis

#### Region-of-interest selection

The amygdala and VS have well-defined anatomical boundaries, so bilateral seeds were created for each region using the Harvard-Oxford subcortical probabilistic atlas amygdala and nucleus accumbens regions thresholded at 50% probability. As our interest in the dACC came from its role in conflict processing, we planned to choose an area of the dACC most closely associated with conflict processing via a meta-analysis of 337 studies involving the term “conflict” on Neurosynth (https://neurosynth.org/analyses/terms/conflict/). To ensure the seed was indeed located in the dACC, we used a conjunction map of the Neurosynth meta-analysis and the Harvard-Oxford cortical probabilistic atlas to pick center coordinates (*x* = 0, *y* = 14, *z* = 34). A 10-mm sphere was created around this center. Seeds were binarized before use.

#### Activation

After preprocessing, participants individual run-level data were analyzed using a fixed-effects general linear model (GLM). Eleven primary regressors were created to represent each event type: Go (“1” at a green), Inhibition (“2” at red), False Alarm (“1” at red), Red Crash (crash following false alarm), Risky (“1” at yellow), Cautious (“2” at yellow), Anticipation (period between risky and feedback), Yellow Crash (crash following risky), Reward (reward following risky), Finish (3s finish line), and Junk (trials of no interest/without responses; Figure 1). To assess trial-by-trial estimates of neural activity during risky decision-making, we used a least-squares all beta-series regression analysis (Rissman et al., 2004) in which one GLM was defined for Risky trials and another for Cautious trials for each run. Trials of interest were separated into their own regressor (resulting in *n* regressors for *n* trials) to achieve an estimate of brain activity (beta value) for every voxel in the brain during each yellow-light decision, where beta values reflect the magnitude of the hemodynamic response evoked by each event. Trials of no interest were represented by one regressor per trial type (e.g., Go, Inhibition, etc.) to preserve the original task design. Events were modeled with a canonical double-gamma hemodynamic response function (HRF) for a variable duration dependent on participant behavior. Rest periods and ITIs were not explicitly modeled and served as the implicit baseline of interest. Temporal derivatives for all regressors, standard and extended motion parameters, and motion outliers were included as covariates of no interest. Standard motion parameters refer to the six rigid-body realignment estimates (three translations: *x, y, z*; and three rotations: pitch, roll, yaw). Extended motion parameters include the temporal derivatives of each parameter and the squared values of both the original and derivative parameters, providing a more comprehensive model of head motion effects in accordance with FSL recommendations. Motion outliers were identified using FSLMotionOutliers, which flags volumes with excessive frame-to-frame displacement or signal change using the box-plot threshold of P75 + 1.5×IQR; these outlier volumes were included as nuisance regressors in the GLM.

Parameter estimates were combined across runs for Risky and Cautious conditions and registered to standard space. Time series were extracted from each region-of-interest (ROI), resulting in beta estimates representing activity in the VS, amygdala, and dACC during each yellow-light decision. Trial-by-trial activation of the VS and amygdala were examined using the lme4 package in R. Two linear mixed-effects models were fit with either VS or amygdala activity as the dependent variable and random intercepts to account for repeated measures within participants. Main effects of and interactions between task condition (Risky vs. Cautious), anxiety, and BAS were tested for each region of interest. Age, sex, and mean relative motion were included as covariates of no interest. We also included an anxiety*BAS*condition interaction term to explore potential moderation effects, consistent with our broader conceptual model. Continuous predictors (e.g., anxiety, BAS, age, motion) were standardized (z-scored) using the base R scale() function prior to inclusion in the models. As we examined neural activity across two regions of interest, we corrected for multiple comparisons by assessing significance as *<(.05/2) = .025, **<(.01/2) = .005, ***<(.001/2) = .0005.

In addition to preregistered analyses of amygdala and VS activation, we conducted an exploratory analysis examining activation in the dACC. The dACC is a central hub within both amygdala–dACC and VS–dACC circuits – the primary circuits investigated in our connectivity analyses – and plays a critical role in conflict monitoring, value-based control, and risk-related decision-making. Because our preregistered analyses focused on connectivity involving the dACC, assessing dACC activation itself was important for interpreting circuit-level findings and understanding how approach motivations and anxiety shape engagement of the broader decision-making network. As this analysis was not preregistered, it should be interpreted with caution and in an exploratory context.

#### Functional connectivity

Beta series correlation analysis (Rissman et al., 2004) was used to examine differences in functional connectivity across task conditions. Timeseries for each ROI were correlated for each condition, resulting in an estimate of VS-dACC and amygdala-dACC functional connectivity for Risky and Cautious decisions for each participant. Correlation values were Fisher transformed for use in group analysis.

Two linear mixed-effects models were fit with either VS-dACC or amygdala-dACC connectivity as the dependent variable and random intercepts to account for repeated measures within participants. Main effects of and interactions between task condition (Risky vs. Cautious), anxiety, and BAS were tested for each circuit of interest. We also included an anxiety*BAS*condition interaction term to explore potential moderation effects, consistent with our broader conceptual model. Age, sex, and mean relative motion were included as covariates of no interest. Continuous predictors (e.g., anxiety, BAS, age, motion) were standardized (*z*-scored) using the base R scale() function prior to inclusion in the models. As we examined functional connectivity across two circuits of interest, we corrected for multiple comparisons by assessing significance as *<(.05/2) = .025, **<(.01/2) = .005, ***<(.001/2) = .0005.

#### RSA: Whole-brain VS functional connectivity

For the exploratory RSA analysis, the VS timeseries was correlated with every other voxel in the brain and Fisher transformed using 3dTcorrelate in AFNI (https://afni.nimh.nih.gov/pub/dist/doc/program_help/3dTcorrelate.html) to generate whole-brain striatal connectivity maps for each subject for each condition. Following this, we used 3ddot in AFNI (https://afni.nimh.nih.gov/pub/dist/doc/program_help/3ddot.html) to compute the correlation coefficient between Risky and Cautious whole-brain VS connectivity maps for each subject. Correlation coefficients were Fisher transformed for group analysis.

#### Resting state

After preprocessing, resting-state time series for each ROI were extracted and correlated, resulting in an estimate of VS-dACC and amygdala-dACC intrinsic functional connectivity for each participant. Correlation values were Fisher transformed for use in group analysis. Two linear regression models were fit with either VS-dACC or amygdala-dACC connectivity as the dependent variable, anxiety and BAS as covariates of interest, and age, sex, and mean relative motion as covariates of no interest. We also included an anxiety*BAS interaction term to explore potential moderation effects, consistent with our broader conceptual model. Continuous predictors (e.g., anxiety, BAS, age, motion) were standardized (*z*-scored) using the base R scale() function prior to inclusion in the models. As we examined functional connectivity across two circuits of interest, we corrected for multiple comparisons by assessing significance as *<(.05/2) = .025, **<(.01/2) = .005, ***<(.001/2) = .0005.

## Results

### Behavioral Results

#### BAS, anxiety, and risk-taking

**Figure 2.**
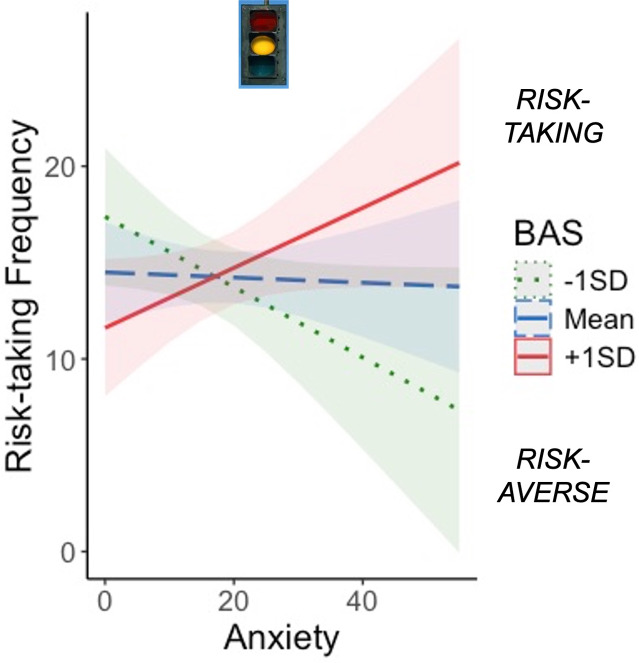
Approach motivations moderate the association between anxiety and task risk-taking. Increasing anxiety was associated with more risk-taking in adolescents with high BAS and risk aversion in adolescents with low BAS (*p* = .012). BAS = behavioral activation system; SD = standard deviation.

Model results are displayed in Table 2. There was a significant effect of sex on task risk-taking frequency such that girls took fewer risks than boys. BAS significantly moderated the effect of anxiety on risk-taking frequency (Figure 2), suggesting that increasing anxiety was associated with more risk-taking among adolescents with high BAS, but less risk-taking among adolescents with low BAS. This supports the idea that approach motivations amplify or buffer anxiety-related risk behavior in adolescents.

**Table 2.** Risk-taking model results. BAS = behavioral activation system

	Risk-Taking Frequency		
*Predictors*	*Estimates*	*CI*	*p*
(Intercept)	0.22	−0.01–0.45	0.061
Anxiety	−0.02	−0.19–0.16	0.834
BAS	0.04	−0.14–0.22	0.641
Age	0.12	−0.06–0.29	0.189
Sex (1 = female)	−0.55	−0.90– −0.20	**0.003**
Anxiety*BAS	0.25	0.06–0.45	**0.012**
Observations	121		
R^2^/R^2^ adjusted	0.141/0.103		

Diagnostic plots of the anxiety*BAS distribution are presented in Figure S1 and demonstrate that moderation was appropriate to test in this sample. Influence diagnostics using Cook’s distance identified 7 cases with values >0.03, which we removed in a sensitivity analysis. Re-running the moderation model without these participants yielded a stronger and more significant anxiety*BAS interaction (*b* = .32, *p* < .001). This confirms the robustness of the interaction and suggests it is not an artifact of outliers or high-anxiety edge cases.

#### BAS, anxiety, and inhibitory control

**Figure 3.**
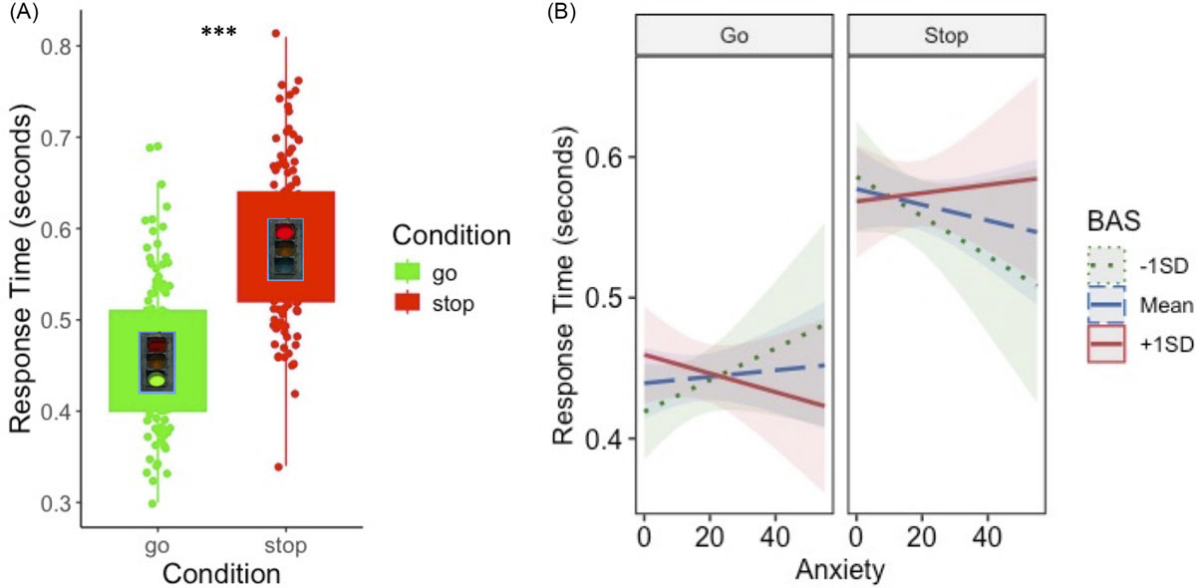
Approach motivations moderate the association between anxiety and task SSRT. (A) Adolescents spent approximately 0.12 s longer inhibiting at red lights than going at green lights (*p* < .001). (B) Increasing anxiety was associated with faster response time when inhibiting a prepotent response in adolescents with low BAS but was associated with slower response time in adolescents with high BAS (*p* < .001). BAS = behavioral activation system; RT = response time; SSRT = stop signal reaction time; SD = standard deviation.

Model results are displayed in Table 3. Participants spent significantly longer stopping at red lights than going at green lights (Figure 3a). There were also significant main effects of sex and age on RT such that girls responded more slowly than boys and older adolescents responded faster than young adolescents. There was a significant anxiety*BAS*condition interaction on RT such that higher anxiety was associated with faster Stop vs. Go RT in adolescents with low BAS but showed the opposite effect in adolescents with high BAS (Figure 3b). Similar to the risk-taking model, this suggests that increasing anxiety sharpens inhibitory control in youth with low BAS but interferes with inhibitory control in youth with high BAS.

**Table 3.** Inhibitory control model results. BAS = behavioral activation system

	Response Time (s)		
*Predictors*	*Estimates*	*CI*	*p*
(Intercept)	−0.15	−0.25– −0.05	0.003
Condition (Stop vs. Go)	0.72	0.66–0.78	<0.001
Anxiety	0.02	−0.06–0.10	0.631
BAS	0.02	−0.07–0.10	0.715
Age	−0.13	−0.20– −0.06	<0.001
Sex (1=female)	0.19	0.05–0.33	0.006
Condition*Anxiety	−0.05	−0.11–0.01	0.106
Condition*BAS	0.03	−0.04–0.09	0.433
Anxiety*BAS	−0.07	−0.16–0.01	0.100
Condition*Anxiety*BAS	0.13	0.06–0.20	<0.001
**Random Effects**			
σ^2^	0.74		
τ_00 Subject_	0.19		
τ_11 Subject. Condition_	0.09		
ρ_01 Subject_	−0.54		
ICC	0.19		
N _Subject_	121		
Observations	27,999		
Marginal R^2^/Conditional R^2^	0.089/0.266		

### fMRI results

#### Task activation

**Figure 4.**
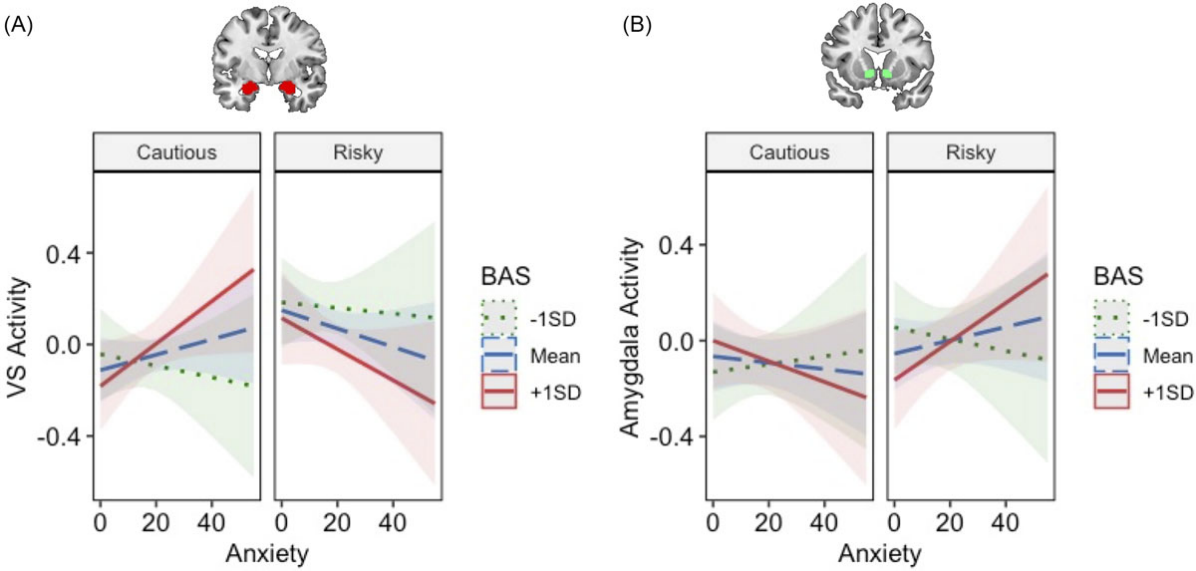
Approach motivations and anxiety show interactive effects on subcortical activity during risk-taking. (A) Higher anxiety was associated with lower VS activation during risky (vs. Cautious) choices among adolescents with higher BAS. (B) Higher anxiety was associated with greater amygdala activity during risky (vs. Cautious) choices among adolescents with higher BAS. BAS = behavioral activation system; VS = ventral striatum; SD = standard deviation.

Across subjects, VS activity was higher when making a Risky versus Cautious choice (*b* = 0.12, *p* = .0003; Table S1). A significant anxiety*BAS*condition interaction emerged (*b* = −0.10, *p* = .006; Figure 4a). Specifically, higher anxiety was associated with lower VS activity during Risky (vs. Cautious) choices among adolescents with higher BAS.

Amygdala activation was also higher during Risky relative to Cautious choices (*b* = 0.10, *p* = .004; Table S2). A significant anxiety*BAS*condition interaction was observed (*b* = 0.10, *p* = .005; Figure 4b). Specifically, higher anxiety was associated with greater amygdala activity during Risky (vs. Cautious) choices among adolescents with higher BAS.

An exploratory model revealed that anxiety was associated with reduced dACC activation across both Risky and Cautious decisions (*b* = −0.11, *p* = .006; Table S3), suggesting diminished engagement of conflict-monitoring or cognitive control systems.

#### Task connectivity

**Figure 5.**
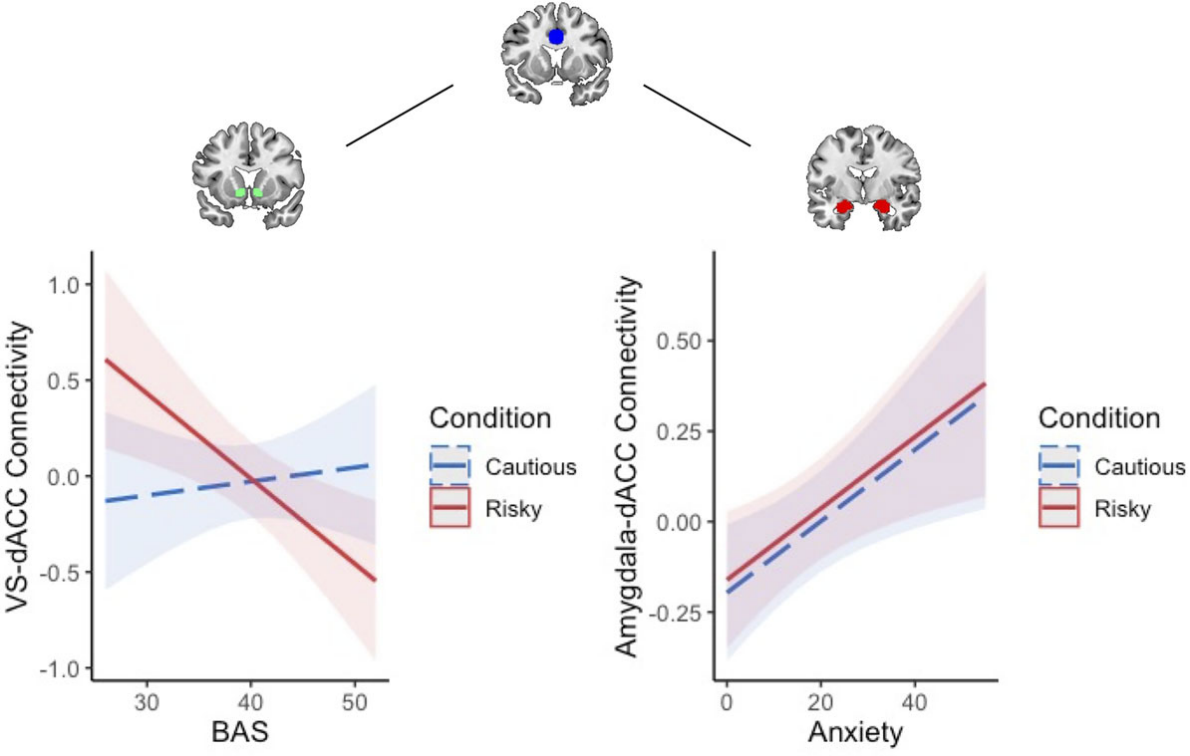
Approach motivations, anxiety, and frontal-subcortical functional connectivity during risk-taking. Adolescents with higher BAS showed reduced VS-dACC connectivity during risk-taking, while adolescents with higher anxiety showed heightened amygdala-dACC connectivity during risk-taking. BAS = behavioral activation system; VS = ventral striatum; dACC=dorsal anterior cingulate cortex; Amy=amygdala.

Driving Game connectivity analyses revealed a significant BAS*condition interaction on VS–dACC connectivity (*b* = −0.30, *p* = .014; Figure 5; Table S4). Higher BAS was associated with reduced VS–dACC coupling during Risky relative to Cautious decisions, suggesting weaker integration between motivational (VS) and regulatory (dACC) signals when adolescents with high BAS engaged in risk-taking.

In contrast, anxiety showed a significant positive association with amygdala–dACC connectivity across both Risky and Cautious decisions (*b* = 0.17, *p* = .01; Figure 4; Table S5), indicating greater limbic-to-regulatory coupling in anxious youth regardless of decision context. Together, these task-based connectivity patterns highlight divergent pathways through which approach motivations and anxiety influence decision-making circuits: BAS was linked to reduced fronto-striatal regulation during risky choices, whereas anxiety was linked to increased fronto-limbic engagement across conditions.

#### Resting-state connectivity

**Figure 6.**
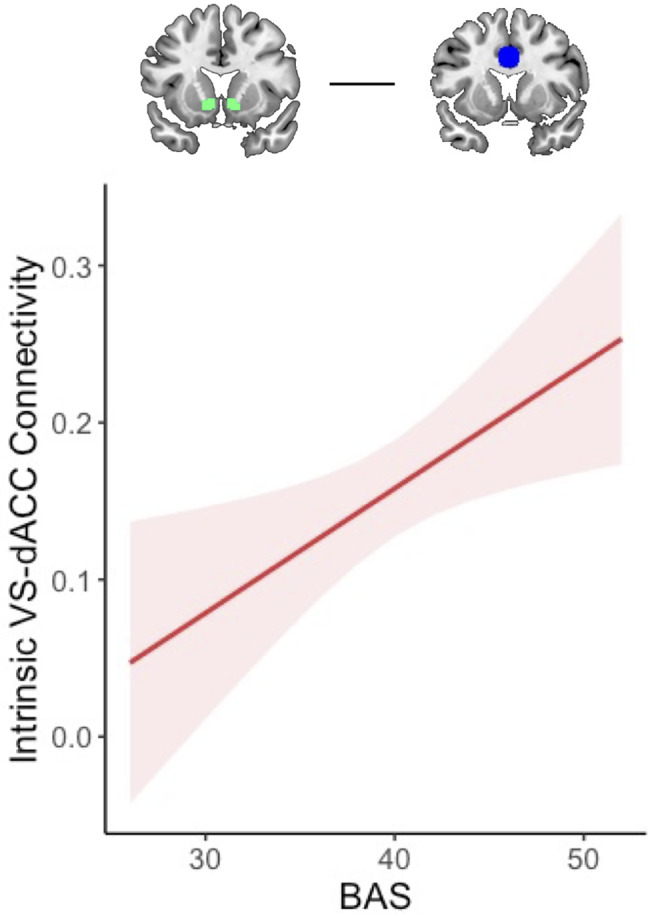
BAS is positively associated with intrinsic VS-dACC connectivity at rest. Increasing BAS sensitivity was associated with increasing intrinsic VS-dACC connectivity. BAS = behavioral activation system; VS = ventral striatum; dACC=dorsal anterior cingulate cortex.

Resting-state analyses revealed a positive association between BAS and intrinsic VS–dACC connectivity (*b* = 0.23, *p* = .012; Figure 6; Table S6), indicating greater tonic fronto-striatal coupling in adolescents with higher approach motivations. In contrast, anxiety was not significantly associated with intrinsic amygdala–dACC connectivity (*b* = −0.04, *p* = .68; Table S7), suggesting that motivational tendencies may exert stronger influence on intrinsic network organization than anxiety symptoms in early adolescence.

However, an exploratory age*anxiety interaction indicated that the normative developmental decrease in amygdala–dACC connectivity was attenuated in youth with higher anxiety (*b* = 0.22, *p* = .005). Older adolescents with elevated anxiety showed less age-related reduction – or more persistent positive coupling – between the amygdala and dACC, a pattern consistent with delayed maturation of emotion-regulatory circuitry.

#### VS dissimilarity

Representational similarity analysis (RSA) revealed that adolescents with higher anxiety showed greater differentiation in striatal connectivity patterns when making Risky versus Cautious decisions (*b* = 0.24, *p* = .01; Figure S2; Table S8). In other words, youth with higher anxiety recruited more distinct multivoxel striatal representations during risky compared with cautious choices, even when this did not translate to reduced risk-taking behavior. BAS was not significantly associated with striatal differentiation (*b* = 0.04, *p* = .65), suggesting that approach motivations did not influence how striatal networks encoded risk versus safety.

## Discussion

This study examined how anxiety and approach motivations (BAS) jointly shape adolescent risk-taking, neural functioning of decision-making circuits during risk-taking, and intrinsic connectivity of decision-making circuits at rest. Consistent with hypotheses, BAS moderated the behavioral effects of anxiety on risk-taking and inhibitory control. When BAS was low, increasing anxiety predicted lower risk-taking and faster inhibition, whereas when BAS was high, increasing anxiety predicted greater risk-taking and slower inhibition. These findings suggest that anxiety does not exert uniform effects on behavior; instead, approach motivations can either buffer or amplify anxiety-related behaviors depending on the adolescent’s motivational profile.

At the neural level, we expected anxiety to map onto heightened amygdala activation and BAS onto heightened VS activation during risky choices. Instead, anxiety and BAS interacted dynamically across reward- and threat-related circuits, paralleling behavioral findings. Specifically, increasing BAS was associated with decreasing VS activation preceding risky choices and increasing VS activation preceding cautious choices – especially among adolescents with higher anxiety. Although high BAS is typically linked to increased striatal responses to reward outcomes (Braams et al., 2015; Schreuders et al., 2018; van Duijvenvoorde et al., 2014), we focused on the decision-making phase to understand the neural processes guiding adolescents’ choices under risk, rather than their responses to outcomes. Reduced VS activation during risky decisions in high-BAS youth may reflect a more automatic, default approach style that bypasses deliberative value comparison, resulting in less striatal engagement. In contrast, choosing cautiously may engage striatal circuits more robustly when it conflicts with an adolescent’s dominant approach tendencies, potentially reflecting the motivational salience or conflict resolution involved in inhibiting a habitual response.

Further, evidence suggests that the striatum plays a central role in avoidance and safety learning, particularly among anxious adolescents (Baker et al., 2023; Delgado et al., 2008; Lago et al., 2017; Levita et al., 2012; Loewke et al., 2021; Rosenberg et al., 2024). Thus, in high-anxiety adolescents, lower VS activation during risky choices may indicate less recruitment of avoidance-related striatal processes. When anxiety is elevated and BAS is high, weakened avoidance signaling coupled with strong approach tendencies may amplify risk-taking behavior, helping reconcile the paradox of blunted VS activation yet heightened approach behavior in high-BAS, high-anxiety youth.

Conversely, higher anxiety was associated with heightened amygdala activation during risk-taking – but again only in youth with high BAS. This pattern suggests that anxiety-related threat vigilance becomes more pronounced precisely in motivational contexts that also heighten approach tendencies. The combination of reduced VS engagement and increased amygdala reactivity indicates a decoupling of reward and threat-related circuits, such that threat salience dominates decision-making while value-differentiation signals in the VS are less engaged. Rather than reflecting reduced reward sensitivity, the blunted VS response in high-BAS anxious youth may index conflict between competing motivational systems – reward seeking versus avoidance – consistent with approach–avoidance conflict models.

Exploratory analyses revealed that anxiety was also associated with reduced dACC activation during both risky and cautious choices, suggesting impaired engagement of cognitive control or conflict monitoring systems – consistent with previous work (Blair et al., 2012). This reduced dACC recruitment may be particularly consequential for high-anxiety, high-BAS youth given their elevated behavioral risk-taking: with weaker conflict monitoring, the brain may be less equipped to integrate competing reward and threat cues, promoting affect-driven or habitual responses over value-informed decisions.

Connectivity analyses supported this interpretation. Consistent with hypotheses, higher BAS was associated with reduced VS–dACC connectivity during risky decisions, suggesting weaker prefrontal regulation of reward circuits during motivated action. In this context, risk-taking in high-BAS youth may reflect diminished integration of striatal and prefrontal control signals rather than exaggerated reward anticipation. Lower VS activation and VS–dACC coupling during risky relative to cautious choices may reflect reduced value differentiation or diminished conflict monitoring between competing action tendencies. This shift in motivational salience away from evaluative processes and toward reflexive responding may further heighten risk-taking in adolescents high in both anxiety and BAS.

In contrast, anxiety was associated with increased amygdala–dACC connectivity during both risky and cautious decisions, possibly reflecting heightened threat-related influence or compensatory monitoring during ambiguous choices. Together, these findings suggest that anxious adolescents may exhibit a dysregulated integration of affective (amygdala), valuation (VS), and control (dACC) systems, characterized by enhanced limbic signaling but reduced recruitment of regulatory regions. This imbalance may contribute to paradoxical behavioral outcomes – such as increased risk-taking despite heightened internal threat sensitivity – especially in high-BAS youth.

Although VS sensitivity is thought to bias adolescent behavior towards approach responses (Ernst, 2014), research suggests that the striatum and its connections with the rest of the brain also play an important role in facilitating and maintaining anxious avoidance (Loewke et al., 2021). In an exploratory analysis quantifying the degree to which striatal networks distinguished between engaging in versus avoiding a risk in the laboratory task, we found that increasing anxiety was associated with greater striatal dissimilarity during risky versus cautious decisions. This supports the idea that adolescents with high anxiety may recruit striatal networks to distinguish risk from safety – even when this does not translate into conservative behavior. Notably, BAS was not associated with these whole-brain similarity patterns, suggesting that anxiety – but not approach motivations – uniquely shapes striatal network configuration to degree of risk, although future work utilizing tasks that vary the degree of risk and reward across conditions will be needed to adequately test this hypothesis.

Resting-state results partially aligned with hypotheses. We predicted that the reduced fronto-striatal connectivity observed in approach-motivated youth during risk-taking would be paralleled by heightened intrinsic fronto-striatal connectivity when the brain is at rest, highlighting the frequent communication between these regions in the absence of a behavioral task in approach-motivated individuals (Adrián-Ventura et al., 2019; Angelides et al., 2017). Indeed, higher BAS was associated with greater intrinsic VS–dACC connectivity, suggesting that fronto-striatal circuits may be more tonically engaged in approach-motivated adolescents. This intrinsic coupling pattern may scaffold the elevated risk-taking observed specifically among high-BAS adolescents with higher anxiety, complementing the reduced task-based connectivity seen during risky decisions.

While previous work has linked anxiety to intrinsic connectivity of the amygdala (Baur et al., 2013; Connolly et al., 2017; Hahn et al., 2011; Roy et al., 2013; Toazza et al., 2016), we did not observe significant main effects of anxiety on intrinsic amygdala-dACC connectivity in this sample. This null finding may be developmentally meaningful given the age range of our sample. During early adolescence, amygdala–prefrontal circuits are undergoing rapid maturation, including shifts from positive to more inverse (regulatory) connectivity patterns between the amygdala and prefrontal regions (Gee et al., 2013, 2016), thought to reflect increasing top-down regulation of emotion and threat responses as youth mature. Indeed, an exploratory analysis revealed that higher anxiety attenuated the typical developmental decrease in amygdala–dACC connectivity. In other words, youth with higher anxiety showed a blunted age-related shift, maintaining stronger or less-negative connectivity between the amygdala and dACC. This may indicate delayed or disrupted maturation of emotion regulation circuitry in youth with higher anxiety, consistent with prior work (Kujawa et al., 2016).

Despite the prevalence of adolescent anxiety disorders, the heterogeneity of their behavior and symptom profiles can make them complex to identify and effectively treat. Adolescents are particularly sensitive to motivational contexts given ongoing brain development and heightened reactivity to both reward and threat (Klein et al., 2020, 2021). Our findings emphasize that anxiety’s behavioral and neural expressions depend critically on motivational context. High-BAS anxious youth appear especially prone to risk-taking and may rely on neural patterns marked by reduced value differentiation (VS), heightened threat monitoring (amygdala), and weakened regulatory integration (dACC and its connectivity). These distinctions have clear clinical implications: high-BAS, high-anxiety youth may be more prone to externalizing behaviors such as impulsive risk-taking or substance use (Nicholls et al., 2014), while low-BAS, high-anxiety youth may show more classic avoidance profiles. On the other hand, high-BAS, high-anxiety youth may achieve greater benefit from exposure-based treatments such as CBT (Norris et al., 2021).

Although this was a community-based sample and not a clinical one, the findings still carry important implications for understanding mechanisms underlying adolescent anxiety. While only a subset of participants reported clinically significant levels of anxiety, the dimensional associations observed suggest that interactions between anxiety symptoms and approach motivations are meaningful even at subclinical levels and may be magnified in clinical populations. For example, anxious adolescents with high BAS in clinical samples may be even more likely to engage in risky behaviors or experience regulatory challenges, while those with low BAS may show more entrenched avoidance patterns. Thus, while the current findings should not be interpreted as diagnostic markers, they may help identify transdiagnostic mechanisms that differentiate subtypes of anxious youth and inform treatment tailoring. Future work using clinical samples and longitudinal designs will be essential for validating these patterns and understanding how they impact mental health trajectories across adolescence.

These findings should be interpreted in the context of several limitations. This study is cross-sectional, limiting the ability to make causal inferences about the effects of anxiety or BAS on risk-taking or neural functioning. Although we uncovered between-person effects based on individual differences, longitudinal tracking will be important for assessing how anxiety and approach motivations develop reciprocally over adolescence. The decision-making nature of the fMRI task also resulted in some participants having fewer than the recommended number of trials in certain conditions; future studies may want to use tasks with more risk-taking trials. While the sample was intentionally broad, relatively few participants had clinically significant anxiety, and effects should be interpreted dimensionally rather than categorically. Finally, while anxiety symptoms and approach motivations showed associations with task-modulated functional connectivity, we did not measure effective connectivity and therefore cannot speak to the direction of communication within our circuits of interest (i.e., whether communication was driven by bottom-up subcortical or top-down frontal influences).

Despite these limitations, this study highlights a dynamic interplay between anxiety and approach motivations in shaping adolescent behavior and neural function. In this early adolescent community sample, high BAS shifted the behavioral expression of anxiety: whereas anxiety is typically associated with avoidance and risk aversion, adolescents with both elevated anxiety and high BAS showed greater risk-taking accompanied by distinct patterns across reward, threat, and control neural systems. These findings suggest that approach motivation does not simply add to anxiety’s effects but can potentiate, reorient, or amplify them, altering how anxious adolescents evaluate and respond to risk. By demonstrating that anxiety’s impact depends on motivational context, this work underscores the need to incorporate motivational traits into transdiagnostic models of adolescent anxiety and highlights a potential mechanism through which some anxious youth may be drawn toward externalizing or risk-prone behaviors. Future longitudinal and clinical studies will be essential for determining whether these neural signatures prospectively mark risk trajectories, signal heightened vulnerability for maladaptive outcomes, or identify subgroups of anxious adolescents who may benefit from tailored intervention strategies.

## Supporting information

10.1017/S0954579426101266.sm001 Baker et al. supplementary material Baker et al. supplementary material

## Data Availability

The data supporting the findings of this study are available from the corresponding author upon reasonable request. **Transparency and Openness (TOP) Statement** **Availability of Data:** Data are available upon reasonable request. **Availability of Code:** Analysis code is available upon reasonable request. **Availability of Materials/Methods:** Study materials and methods are described in the manuscript and supplementary materials.

## Supplementary material

The supplementary material for this article can be found at https://doi.org/10.1017/S0954579426101266.

## Preprint statement

This paper is posted on PsyArXiv as a preprint.

**Preprint link:** https://osf.io/nqdxc

**Preprint citation:** Baker, A. E., Padgaonkar, N. T., Peris, T. S., & Galván, A. (2022, August 24). Anxiety symptoms interact with approach motivations in adolescent risk-taking. https://doi.org/10.31234/osf.io/nqdxc

## Pre-registration statement

Study hypotheses were pre-registered on the Open Science Framework.

**Active link:** https://osf.io/qebd6

**Date stamped:** Mar 3, 2022, 12:31 PM

**Deviations:** We conducted exploratory analyses in the brain data to probe interactions between anxiety and BAS, an exploratory analysis probing interactions between anxiety and age, and an exploratory RSA analysis. All exploratory analyses were clearly marked as such.
