# Supplementary material for: Anxiety symptoms interact with approach motivations in adolescent risk-taking
Source: Dev Psychopathol. 2026 Feb 24:1–14. Online ahead of print. doi: 10.1017/S0954579426101266 (PMC12957653; doi:10.1017/S0954579426101266)
Supplement: Baker et al. supplementary material [file S0954579426101266sup001.docx]

**Figure S1. Diagnostic plots of the association between anxiety and BAS.** BAS = behavioral activation system.

**
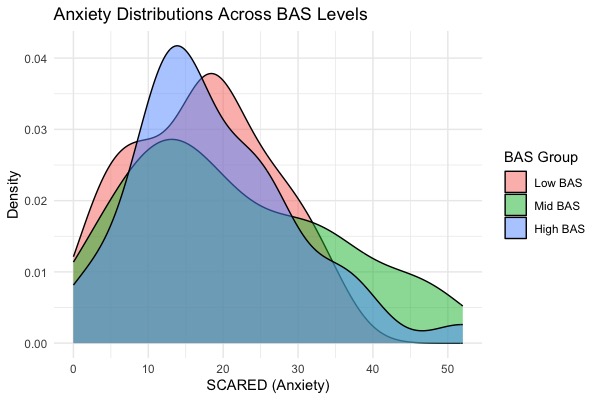

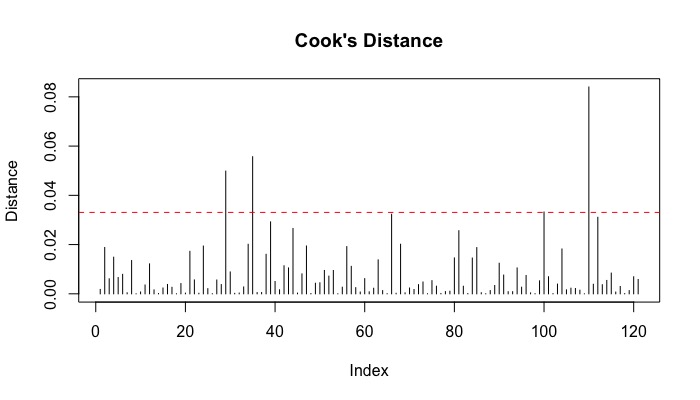

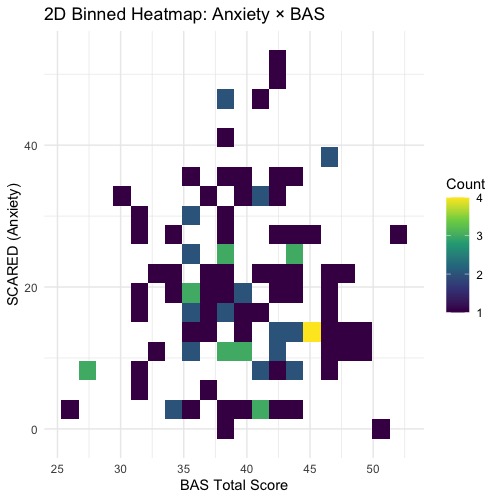

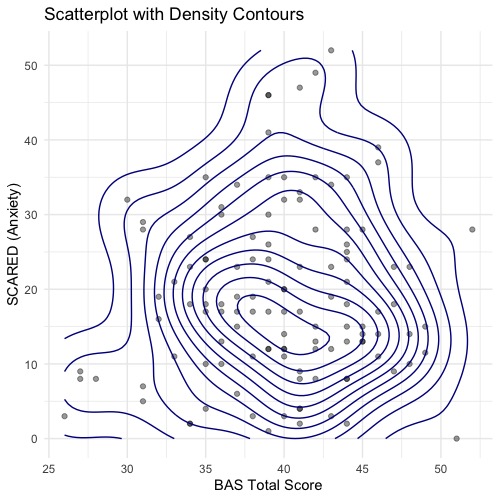
**

**Table S1. VS activation model results.** VS = ventral striatum; BAS = behavioral activation system.

|  | **VS Activation** | | |
| --- | --- | --- | --- |
| *Predictors* | *Estimates* | *CI* | *p* |
| (Intercept) | -0.02 | -0.12 – 0.07 | 0.645 |
| Condition (Risky vs. Cautious) | 0.12 | 0.06 – 0.19 | **<0.001** |
| Anxiety | 0.04 | -0.04 – 0.11 | 0.303 |
| BAS | 0.04 | -0.03 – 0.12 | 0.283 |
| Age | -0.01 | -0.08 – 0.06 | 0.784 |
| Sex (1=female) | -0.06 | -0.20 – 0.08 | 0.383 |
| Mean Relative Motion | -0.02 | -0.08 – 0.05 | 0.603 |
| Condition*Anxiety | -0.08 | -0.15 – -0.02 | **0.012** |
| Condition*BAS | -0.13 | -0.20 – -0.06 | **<0.001** |
| Anxiety*BAS | 0.07 | -0.02 – 0.15 | 0.113 |
| Condition*Anxiety*BAS | -0.10 | -0.17 – -0.03 | **0.006** |
| **Random Effects** | | | |
| σ^2^ | 0.89 | | |
| τ_00_ _Subject_ | 0.11 | | |
| ICC | 0.11 | | |
| N _Subject_ | 121 | | |
| Observations | 3847 | | |
| Marginal R^2^ / Conditional R^2^ | 0.013 / 0.119 | | |

**Table S2. Amygdala activation model results.** BAS = behavioral activation system

|  | **Amygdala Activation** | | |
| --- | --- | --- | --- |
| *Predictors* | *Estimates* | *CI* | *p* |
| (Intercept) | -0.09 | -0.19 – 0.01 | 0.081 |
| Condition (Risky vs. Cautious) | 0.10 | 0.03 – 0.16 | **0.004** |
| Anxiety | -0.01 | -0.09 – 0.06 | 0.768 |
| BAS | 0.01 | -0.07 – 0.09 | 0.804 |
| Age | -0.03 | -0.10 – 0.04 | 0.461 |
| Sex (1=female) | 0.09 | -0.05 – 0.24 | 0.198 |
| Mean Relative Motion | -0.07 | -0.14 – -0.01 | 0.029 |
| Condition*Anxiety | 0.05 | -0.02 – 0.11 | 0.162 |
| Condition*BAS | -0.02 | -0.09 – 0.05 | 0.508 |
| Anxiety*BAS | -0.04 | -0.12 – 0.05 | 0.408 |
| Condition*Anxiety*BAS | 0.10 | 0.03 – 0.17 | **0.005** |
| **Random Effects** | | | |
| σ^2^ | 0.87 | | |
| τ_00_ _Subject_ | 0.12 | | |
| ICC | 0.12 | | |
| N _Subject_ | 121 | | |
| Observations | 3847 | | |
| Marginal R^2^ / Conditional R^2^ | 0.015 / 0.131 | | |

**Table S3. dACC activation model results.** dACC = dorsal anterior cingulate; BAS = behavioral activation system**.**

|  | **dACC Activation** | | |
| --- | --- | --- | --- |
| *Predictors* | *Estimates* | *CI* | *p* |
| (Intercept) | 0.03 | -0.07 – 0.13 | 0.578 |
| Condition (Risky vs. Cautious) | -0.08 | -0.14 – -0.01 | **0.023** |
| Anxiety | -0.11 | -0.19 – -0.03 | **0.006** |
| BAS | -0.03 | -0.11 – 0.06 | 0.537 |
| Age | 0.03 | -0.05 – 0.10 | 0.491 |
| Sex (1=female) | -0.02 | -0.17 – 0.13 | 0.827 |
| Mean Relative Motion | -0.08 | -0.15 – -0.01 | **0.029** |
| Condition*Anxiety | 0.08 | 0.01 – 0.14 | **0.026** |
| Condition*BAS | 0.03 | -0.03 – 0.10 | 0.305 |
| Anxiety*BAS | 0.07 | -0.02 – 0.16 | 0.148 |
| Condition*Anxiety*BAS | 0.03 | -0.04 – 0.10 | 0.347 |
| **Random Effects** | | | |
| σ^2^ | 0.86 | | |
| τ_00_ _Subject_ | 0.13 | | |
| ICC | 0.13 | | |
| N _Subject_ | 121 | | |
| Observations | 3847 | | |
| Marginal R^2^ / Conditional R^2^ | 0.021 / 0.149 | | |

**Table S4. Task-based VS-dACC connectivity model results.** VS = ventral striatum; dACC = dorsal anterior cingulate; BAS = behavioral activation system. *NOTE:* The interaction between Condition and BAS survived multiple comparison correction after non-significant interactions were removed from the model (*b*=-.30, *p*=.014).

|  | **VS-dACC Connectivity** | | |
| --- | --- | --- | --- |
| *Predictors* | *Estimates* | *CI* | *p* |
| (Intercept) | -0.04 | -0.26 – 0.18 | 0.704 |
| Condition (Risky vs. Cautious) | 0.03 | -0.21 – 0.27 | 0.812 |
| Anxiety | 0.04 | -0.15 – 0.22 | 0.702 |
| BAS | 0.04 | -0.15 – 0.23 | 0.685 |
| Age | -0.07 | -0.20 – 0.07 | 0.341 |
| Sex (1=female) | 0.06 | -0.22 – 0.34 | 0.671 |
| Mean Relative Motion | -0.00 | -0.14 – 0.13 | 0.954 |
| Condition*Anxiety | -0.07 | -0.32 – 0.17 | 0.547 |
| Condition*BAS | -0.29 | -0.55 – -0.03 | **0.027** |
| Anxiety*BAS | -0.00 | -0.21 – 0.20 | 0.971 |
| Condition*Anxiety*BAS | 0.03 | -0.24 – 0.31 | 0.811 |
| **Random Effects** | | | |
| σ^2^ | 0.92 | | |
| τ_00_ _Subject_ | 0.08 | | |
| ICC | 0.08 | | |
| N _Subject_ | 121 | | |
| Observations | 242 | | |
| Marginal R^2^ / Conditional R^2^ | 0.042 / 0.120 | | |

**Table S5. Task-based amygdala-dACC connectivity model results.** dACC = dorsal anterior cingulate; BAS = behavioral activation system. *NOTE:* The association between anxiety and amygdala-dACC connectivity was significant after non-significant interactions were removed from the model (*b*=.17, *p*=.01).

|  | **Amygdala-dACC Connectivity** | | |
| --- | --- | --- | --- |
| *Predictors* | *Estimates* | *CI* | *p* |
| (Intercept) | 0.08 | -0.13 – 0.29 | 0.463 |
| Condition (Risky vs. Cautious) | 0.06 | -0.19 – 0.30 | 0.655 |
| Anxiety | 0.13 | -0.05 – 0.31 | 0.154 |
| BAS | 0.06 | -0.13 – 0.25 | 0.520 |
| Age | -0.07 | -0.20 – 0.07 | 0.328 |
| Sex (1=female) | -0.24 | -0.50 – 0.03 | 0.086 |
| Mean Relative Motion | -0.15 | -0.28 – -0.02 | 0.028 |
| Condition*Anxiety | 0.09 | -0.15 – 0.34 | 0.443 |
| Condition*BAS | -0.26 | -0.52 – -0.01 | 0.045 |
| Anxiety*BAS | -0.07 | -0.27 – 0.13 | 0.478 |
| Condition*Anxiety*BAS | 0.04 | -0.23 – 0.32 | 0.748 |
| **Random Effects** | | | |
| σ^2^ | 0.91 | | |
| τ_00_ _Subject_ | 0.06 | | |
| ICC | 0.06 | | |
| N _Subject_ | 121 | | |
| Observations | 242 | | |
| Marginal R^2^ / Conditional R^2^ | 0.075 / 0.129 | | |

**Table S6. Resting-state VS-dACC connectivity model results.** VS = ventral striatum; dACC = dorsal anterior cingulate; BAS = behavioral activation system.

|  | **VS-dACC Connectivity** | | |
| --- | --- | --- | --- |
| *Predictors* | *Estimates* | *CI* | *p* |
| (Intercept) | 0.16 | -0.08 – 0.41 | 0.188 |
| Anxiety | -0.01 | -0.19 – 0.16 | 0.884 |
| BAS | 0.23 | 0.05 – 0.41 | **0.012** |
| Age | -0.07 | -0.25 – 0.11 | 0.456 |
| Sex (1=female) | -0.34 | -0.70 – 0.02 | 0.060 |
| Mean Relative Motion | 0.04 | -0.14 – 0.22 | 0.649 |
| Anxiety*BAS | 0.02 | -0.17 – 0.21 | 0.803 |
| Observations | 124 | | |
| R^2^ / R^2^ adjusted | 0.098 / 0.051 | | |

**Table S7. Resting-state amygdala-dACC connectivity model results.** dACC = dorsal anterior cingulate; BAS = behavioral activation system.

|  | **Amygdala-dACC Connectivity** | | |
| --- | --- | --- | --- |
| *Predictors* | *Estimates* | *CI* | *p* |
| (Intercept) | 0.17 | -0.08 – 0.42 | 0.178 |
| Anxiety | -0.04 | -0.22 – 0.14 | 0.680 |
| BAS | 0.07 | -0.11 – 0.26 | 0.434 |
| Age | -0.16 | -0.34 – 0.02 | 0.077 |
| Sex (1=female) | -0.35 | -0.70 – 0.01 | 0.059 |
| Mean Relative Motion | 0.07 | -0.11 – 0.26 | 0.421 |
| Anxiety*BAS | -0.04 | -0.23 – 0.15 | 0.686 |
| Observations | 124 | | |
| R^2^ / R^2^ adjusted | 0.082 / 0.035 | | |

**Table S8. VS dissimilarity model results.** VS = ventral striatum; BAS = behavioral activation system.

|  | **VS Dissimilarity** | | |
| --- | --- | --- | --- |
| *Predictors* | *Estimates* | *CI* | *p* |
| (Intercept) | -0.10 | -0.34 – 0.14 | 0.390 |
| Anxiety | 0.24 | 0.06 – 0.42 | **0.010** |
| BAS | 0.04 | -0.14 – 0.23 | 0.651 |
| Age | -0.11 | -0.29 – 0.07 | 0.235 |
| Sex (1=female) | 0.25 | -0.12 – 0.62 | 0.179 |
| Mean Relative Motion | 0.06 | -0.13 – 0.24 | 0.547 |
| Anxiety*BAS | -0.08 | -0.28 – 0.12 | 0.445 |
| Observations | 121 | | |
| R^2^ / R^2^ adjusted | 0.097 / 0.049 | | |

**Figure S2. Anxiety is associated with more dissimilarity in VS connectivity patterns when making Risky versus Cautious choices.** Adolescents with higher anxiety show more neural differentiation in whole-brain striatal connectivity patterns between Risky vs. Cautious choices, suggesting that striatal connectivity may encode different aspects of decision situations depending on youth anxiety. VS = ventral striatum; BAS = behavioral activation system.

**
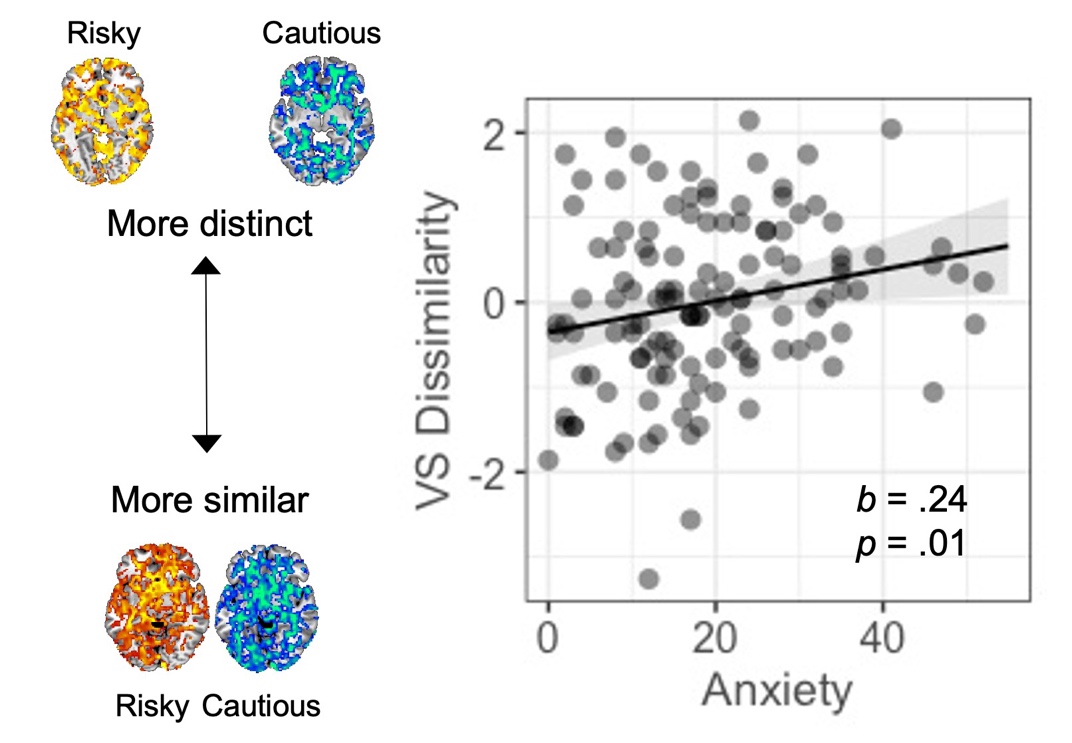
**
